# Impact of filgotinib on sacroiliac joint magnetic resonance imaging structural lesions at 12 weeks in patients with active ankylosing spondylitis (TORTUGA trial)

**DOI:** 10.1093/rheumatology/keab543

**Published:** 2021-08-05

**Authors:** Walter P Maksymowych, Mikkel Østergaard, Robert Landewé, William Barchuk, Ke Liu, Chantal Tasset, Leen Gilles, Thijs Hendrikx, Robin Besuyen, Xenofon Baraliakos

**Affiliations:** 1 Department of Medicine, University of Alberta, Edmonton, AB, Canada; 2 Copenhagen Center for Arthritis Research, Center for Rheumatology and Spine Diseases, Rigshospitalet, Glostrup; 3 Department of Clinical Medicine, University of Copenhagen, Copenhagen, Denmark; 4 Department of Rheumatology, Amsterdam University Medical Center, Amsterdam and Zuyderland Medical Center, Heerlen, The Netherlands; 5 Clinical Research, Gilead Sciences, Foster City, CA, USA; 6 Late Stage Development, Galapagos NV; 7 Biostatistics, LACO, Contracted by Galapagos NV, Mechelen, Belgium; 8 Medical Affairs; 9 Clinical Development, Galapagos BV, Leiden, The Netherlands; 10 Rheumazentrum Ruhrgebiet Herne, Ruhr-University Bochum, Bochum, Germany

**Keywords:** ankylosing spondylitis, filgotinib, inflammation, magnetic resonance imaging, sacroiliac joint, therapeutics

## Abstract

**Objective:**

To assess the effect of filgotinib, which preferentially inhibits Janus kinase 1 (JAK1), on MRI measures of structural change in the SI joint in patients with active AS in the TORTUGA trial.

**Methods:**

Adults with active AS and inadequate response/intolerance to two or more NSAIDs were randomized 1:1 to filgotinib 200 mg (*n* = 58) or placebo (*n* = 58) once daily for 12 weeks. In this post hoc analysis, T1-weighted MRI scans of the SI joint were evaluated by two independent readers using Spondyloarthritis Research Consortium of Canada (SPARCC) Sacroiliac Joint Structural Score (SSS) definitions for erosion, backfill, fat metaplasia and ankylosis. Correlations between SPARCC SSS and improvement in clinical outcomes were also assessed.

**Results:**

MRI scans from 87 patients (48 filgotinib, 39 placebo) were evaluated. At baseline there were no notable differences between filgotinib and placebo for any MRI structural lesion types. From baseline to week 12, filgotinib was associated with a significant reduction in SI joint erosion score (*P* = 0.02) and an increase in backfill score (*P* = 0.005) *vs* placebo, with no significant between-group differences for ankylosis (*P* = 0.46) or fat metaplasia (*P* = 0.17). At week 12, the change in SPARCC MRI SI joint inflammation scores correlated positively with erosion scores but negatively with backfill scores.

**Conclusion:**

The significant changes in MRI structural lesions induced by filgotinib in the SI joint by week 12 demonstrate that tissue repair can be observed very soon after starting treatment with a JAK1 preferential inhibitor. This could have prognostic implications for development of ankylosis.

**Trial registration:**

ClinicalTrials.gov, http://clinicaltrials.gov, NCT03117270

Rheumatology key messages

- The JAK inhibitor filgotinib was associated with MRI structural benefits in patients with AS.
- SPARCC SI joint erosion and backfill significantly decreased and increased, respectively, with filgotinib *vs* placebo.
- Structural changes occurred within the first 12 weeks of treatment, correlating with a reduction in inflammation.

## Introduction

AS is a chronic immune-mediated condition characterized by inflammation in the axial skeleton, with early involvement of the sacroiliac (SI) joint and spine [1, 2]. Despite erosive changes in the SI joint at disease onset, more advanced stages involve new bone formation and joint fusion [3]. AS is associated with chronic pain and progressive structural changes leading to impaired physical function and reduced quality of life [3–5].

MRI is the only imaging modality that can directly detect both inflammatory and structural changes in the SI joint and spine and thus is useful for the early assessment of AS [6–10]. Fat-suppressed MRI sequences [e.g. short tau inversion recovery (STIR)] detect active inflammatory lesions [e.g. bone marrow oedema (BMO)/osteitis], while T1-weighted (T1W) MRI sequences can identify structural changes [10].

AS is sometimes referred to as radiographic or axial spondyloarthritis (r-axSpA or axSpA, respectively). Although the terms are based on different criteria [modified New York criteria and Assessment of Spondylo Arthritis international Society (ASAS) criteria, respectively], they represent largely overlapping populations [11].

The ASAS recently updated definitions for SI joint structural MRI lesions, which included erosion, fat lesions, fat metaplasia in an erosion cavity (also known as backfill), ankylosis and non-bridging bone bud [12]. Erosion, which includes both a breach in cortical bone and loss of adjacent marrow matrix, is a common structural lesion and can be detected on MRI with comparable reliability to subchondral inflammation [12]. Ankylosis occurs after erosion repair, and fat metaplasia and backfill are considered key intermediaries in the development of SI joint ankylosis [13]. After inflammation resolves, fat metaplasia—replacement of an inflammatory lesion in the bone marrow with new tissue—can occur [12]. In an erosion cavity, this is called backfill. Backfill is identified on T1W scans comprising two components: a bright signal within the erosion cavity indicating reparative formation of new tissue in the cavity and an irregular band of black signal indicating sclerosis at the border of the original erosion. It can be reliably detected on MRI [12]. Although not yet included in the ASAS definition of a positive MRI for classification of axSpA (which is based solely on the presence of BMO) [14], MRI structural lesions occur frequently in patients with the condition [15].

Until recently, treatment for patients whose AS persisted despite physiotherapy and continuous NSAIDs comprised TNF inhibitors [16]. These agents reduced inflammation, reduced erosions and increased backfill in 12 week r-axSpA and non-radiographic axSpA studies [17, 18]. Understanding of the immunopathology of AS, in particular the IL-23–IL-17 axis, has paved the way for newer biologics, and the approval of IL-17 inhibitors has been a major advance [3, 19]. Janus kinases (JAKs) directly and indirectly mediate cytokine signalling across multiple inflammatory pathways implicated in AS pathogenesis, thus the JAK pathway is a potential therapeutic target [20]. The JAK inhibitor tofacitinib, which preferentially inhibits JAK3 and/or JAK1, thus interfering with the IL-17, IL-21 and IL-23 inflammatory cascade [21], has shown a clinical effect in AS [22]. Similarly, filgotinib, which provides preferential inhibition of JAK1, was shown to reduce disease activity and improve symptoms in patients with active AS in the phase 2 TORTUGA trial, decreasing inflammation of the SI joint and spine, as indicated by MRI [23]. The JAK inhibitor upadacitinib also demonstrated efficacy in a phase 2/3 study [24]. However, the effects of IL-17 and JAK inhibitors on structural lesions are unknown.

We present a post hoc analysis of the effects of filgotinib on MRI measures of structural changes in the SI joint of patients from the TORTUGA trial. MRIs were reassessed using the Spondyloarthritis Research Consortium of Canada (SPARCC) Sacroiliac Joint Structural Score (SSS), a validated method to assess the structural lesions of erosion, backfill, fat metaplasia and ankylosis on T1W MRI scans [25].

## Methods

### TORTUGA study design

TORTUGA (NCT03117270) was a multicentre, placebo-controlled, double-blind, randomized trial. The study design has been reported previously [23]. Briefly, 116 adults from seven countries (Belgium, Bulgaria, Czech Republic, Estonia, Poland, Spain and Ukraine) with active AS and inadequate response or intolerance to two or more NSAIDs were randomized 1:1 to oral filgotinib 200 mg (*n* = 58) or placebo (*n* = 58) once daily for 12 weeks. Previous use of one TNF inhibitor was permitted (limited to 30% of enrolled patients). The study protocol was reviewed and approved by the central or individual independent ethics committee in each participating country (Supplementary Table S1, available at *Rheumatology* online).

MRI scans were taken at baseline and week 12 (or at an early discontinuation visit). Changes in BMO from baseline were assessed in an independent reading campaign using SPARCC MRI SI joint and spine inflammation scoring methods applied to SI joint and spine MRI STIR scans [26, 27]. MRI scans taken at baseline and week 12 (or at an early discontinuation visit) were reviewed by two independent readers who were unaware of the time point at which the scan had been taken. The change in BMO was the mean score of the two independent readers, or for cases requiring adjudication, the mean score of the adjudicator and the closest score of the two primary readers.

### Assessment of structural changes

In this post hoc analysis, T1W MRI scans of the SI joint from the TORTUGA trial that were originally evaluated for SPARCC SI joint inflammation were re-evaluated using standardized SPARCC SSS definitions [25]. While the SPARCC SI joint inflammation score evaluates the presence, depth and intensity of bone marrow inflammation, the SPARCC SSS assesses the structural lesions of erosion, backfill, fat metaplasia and ankylosis in the SI joint. MRI scans were scored by two independent experts who were blinded to the time point, assigned treatment and spine images (and were read independently of STIR scans). According to the SPARCC SSS method, the presence of structural lesions was recorded in SI joint quadrants for erosion and fat metaplasia and in SI joint halves for backfill and ankylosis on five consecutive semi-coronal slices, starting from the transitional slice and scrolling anteriorly, using direct online data entry based on a schematic of the SI joint. Scoring for the five slices ranged from 0 to 40 for erosion and fat metaplasia and from 0 to 20 for backfill and ankylosis. Backfill and erosion were mutually exclusive. Primary readers and the adjudicator completed a standardized and validated real-time iterative calibration module (available at www.carearthritis.com/mriportal/sss/index/) for the SPARCC SSS score before assessment of the TORTUGA MRI scans [28].

Endpoints included the change from baseline in SPARCC SSS total erosion, total backfill, total fat metaplasia and total ankylosis scores at week 12; the proportion of patients with a decrease, increase or no change in structural lesion score (erosion, backfill, ankylosis and fat metaplasia) at week 12, according to the baseline SPARCC BMO; correlation between changes in structural lesions and the change in clinical parameters; and the number of patients with erosion who developed backfill at week 12.

### Statistical analysis

All patients with MRI scans with one or more measurement at both baseline and week 12 or an early discontinuation visit during the TORTUGA trial were included. Observed changes from baseline were evaluated using analysis of covariance with factors for treatment, baseline value and randomization stratification by prior TNF inhibitor use. Least squares mean changes from baseline and between-group differences with 95% CIs were calculated. Cumulative probability plots were generated to compare the change from baseline to week 12 for each structural lesion for the treatment groups.

The mean of the two reader scores was used to compare the change in structural lesion scores between treatment groups. Interreader intraclass correlation coefficients (ICCs) were calculated to assess the consistency and reliability of scoring between the two MRI readers using the ICC 2.1 model. As pre-specified, interreader discrepancies were resolved by a third independent adjudicator if the change from baseline in SPARCC SSS for structural change measurements for any domain differed by ≥4 points in different directions (one reader detected an improvement, the other detected a worsening) or ≥6 points in the same direction (both detected either improvement or worsening). For cases requiring adjudication, the final score was derived from the mean of the adjudicator’s score and the closest score of the two primary readers.

An ordered logistic regression model tested for treatment differences among categories of change from baseline to week 12 (i.e. decreased, increased, no change) for each structural lesion type, according to baseline SPARCC BMO ≥2 or <2. A SPARCC MRI SI joint score ≥2 is an established indicator of SI joint inflammation on MRI [29]. Pearson correlations were determined between the change from baseline in structural lesions at week 12 and the change in the following: CRP, Ankylosing Spondylitis Disease Activity Score (ASDAS), BASDAI, BASFI, SPARCC MRI SI joint inflammation and SPARCC MRI spine 23-discovertebral units inflammation score.

The TORTUGA trial was not powered for comparison of these post hoc endpoints; all *P*-values should be considered nominal.

## Results

### Patients

MRI scans from 87 patients (48 filgotinib, 39 placebo) with an evaluable MRI at both baseline and week 12 (or an early discontinuation visit) were re-evaluated in this post hoc analysis. Disease activity was similar between these patients and the 29 patients from the TORTUGA trial who were excluded because of missing MRI scans (Supplementary Table S2, available at *Rheumatology* online). In most cases, MRI scans were missing because patients were unavailable or unwilling to undergo the MRI scan at the week 12/early discontinuation visit. Of patients included in the current analysis, there were no notable differences in demographics or baseline disease characteristics, including SPARCC SSS scores, between the filgotinib and placebo groups (Table 1). The total ankylosis score (range 0–20) was >15 in approximately one-third of patients, indicating advanced disease. Eight patients (9.2%) had received prior TNF inhibitor therapy [4 (8.3%) in the filgotinib group and 4 (10.3%) in the placebo group].

**Table 1:** Demographics and baseline disease characteristics of patients with an MRI scan at both baseline and week 12 (or early discontinuation)

Characteristics	Filgotinib (*n* = 48)	Placebo (*n* = 39)	All (*N* = 87)
Age, years, mean (s.d.)	40.7 (11.47)	41.9 (9.28)	41.2 (10.50)
Male, *n* (%)	37 (77.1)	28 (71.8)	65 (74.7)
Weight, kg, mean (s.d.)	74.3 (11.36)	76.4 (16.93)	75.2 (14.08)
BMI, kg/m^2^, mean (s.d.)	25.1 (3.53)	25.9 (3.95)	25.5 (3.72)
Time from symptom onset, years, mean (s.d.)	7.0 (1.14)	7.0 (1.35)	7.0 (1.24)
HLA-B27 positivity, *n* (%)	41 (95.3)	33 (91.7)	74 (93.7)
ASDAS, mean (s.d.)	4.3 (0.57)	4.1 (0.73)	4.2 (0.64)
BASDAI, mean (s.d.)	7.0 (1.14)	7.0 (1.35)	7.0 (1.23)
BASFI, mean (s.d.)	7.0 (1.53)	6.8 (1.59)	6.9 (1.55)
BASMI, linear, mean (s.d.)	4.9 (1.63)	5.2 (1.69)	5.1 (1.65)
hs-CRP, mg/l, mean (s.d.)	19.8 (13.25)	19.5 (16.73)	19.7 (14.82)
hs-CRP ≥ ULN,^a^*n* (%)	35 (72.9)	23 (59.0)	58 (66.7)
MRI SPARCC spine, mean (s.d.)	20.6 (20.54)	16.4 (21.58)	18.7 (20.99)
MRI SPARCC SI joint, mean (s.d.)	7.7 (11.52)	4.9 (6.40)	6.4 (9.62)
Enthesitis at baseline,^b^*n* (%)	39 (84.8)	30 (76.9)	69 (81.2)
Total SPARCC SSS ankylosis score at baseline,^c^*n* (%)			
<10	59 (50.0)	26 (44.8)	55 (47.4)
10–15	7 (12.1)	7 (12.1)	14 (12.1)
>15	20 (34.5)	18 (31.0)	38 (32.8)
SPARCC SSS erosion score (range 0–40), mean (s.d.)	3.4 (5.34)	2.6 (3.76)	3.0 (4.69)
SPARCC SSS backfill score (range 0–20), mean (s.d.)	1.0 (1.99)	1.4 (2.59)	1.2 (2.27)
SPARCC SSS ankylosis score (range 0–20), mean (s.d.)	9.6 (8.15)	9.8 (8.45)	9.7 (8.24)
SPARCC SSS fat metaplasia score (range 0–40), mean (s.d.)	4.2 (6.06)	4.4 (5.44)	4.3 (5.76)
MASES, mean (s.d.)	4.2 (3.1)	3.4 (3.2)	3.8 (3.13)
csDMARD use, *n* (%)	18 (37.5)	16 (41.0)	34 (39.1)
Methotrexate	6 (12.5)	3 (7.7)	9 (10.3)
Sulfasalazine (oral)	12 (25.0)	13 (33.3)	25 (28.7)
NSAID use, *n* (%)	36 (75.0)	25 (64.1)	61 (70.1)
Glucocorticosteroid use, *n* (%)	5 (10.4)	8 (20.5)	13 (14.9)
Previous TNF inhibitor therapy, *n* (%)	4 (8.3)	4 (10.3)	8 (9.2)

a The ULN for hs-CRP is 10 mg/l.

b Data shown for patients with one or more tender enthesis at baseline.

c Data missing for two patients in the filgotinib 200 mg treatment group and seven patients on placebo. csDMARD: conventional synthetic DMARD; hs-CRP: high-sensitivity CRP; MASES: Maastricht Ankylosing Spondylitis Enthesitis Score; ULN: upper limit of normal.

### Changes in structural lesions

**Fig. 1:**
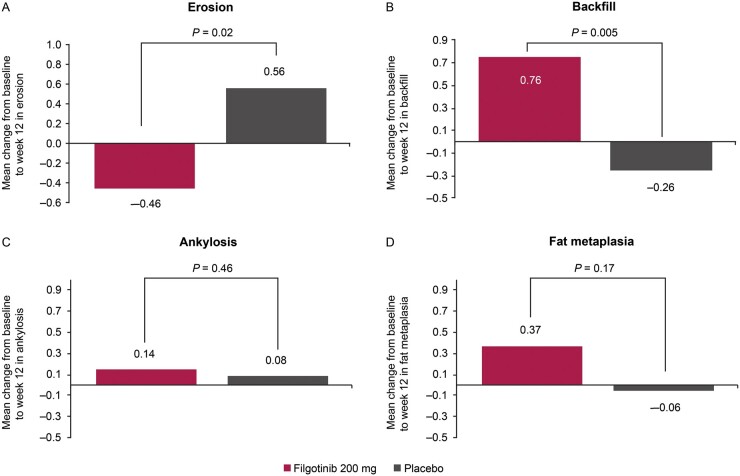
Mean change from baseline to week 12 in the (**A**) total erosion score, (**B**) total backfill score, (**C**)total ankylosis score (**D**) and fat metaplasia.

**Fig. 2:**
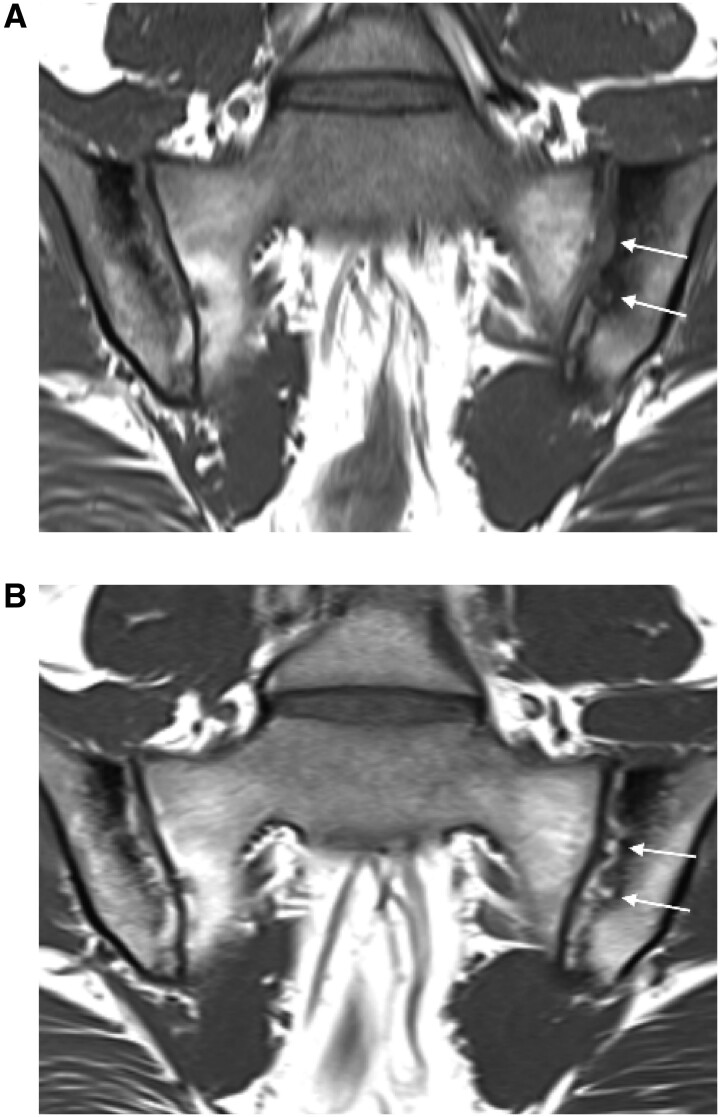
Illustrative T1W MRI scans at (**A**) baseline and (**B**) week 12 from a patient who received filgotinib In (A) the arrows point to an extensive erosion of the left iliac bone on the T1W MRI scan. In (B) the arrows demonstrate the appearance of bright tissue filling in the cavity of the erosion, bordered by an irregular dark band. This is the characteristic appearance of backfill.

The mean change from baseline to week 12 in SPARCC SSS for erosion and backfill was significantly different for filgotinib and placebo (Fig. 1; Supplementary Table S3, available at *Rheumatology* online). Erosion scores decreased in the filgotinib group and increased in the placebo group (*P* = 0.02 for between-group difference). Backfill scores increased in the filgotinib group but not in the placebo group (*P* = 0.005). An example of backfill development during 12 weeks of filgotinib treatment is shown in Fig. 2.

**Fig. 3:**
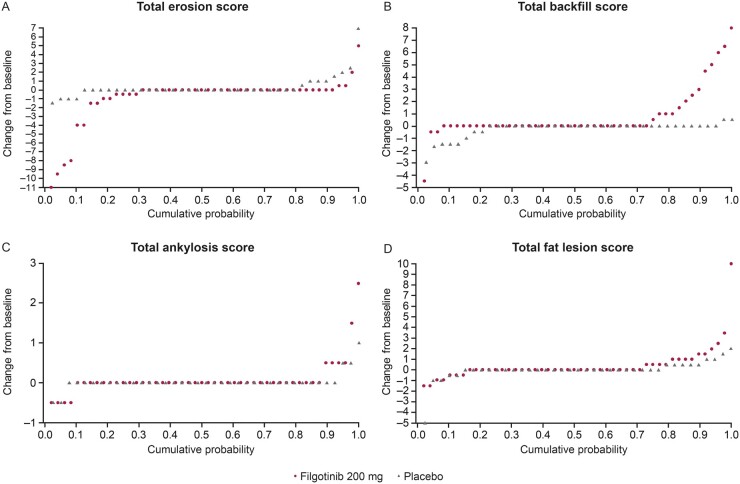
Cumulative probability of change from baseline to week 12 in (**A**) total erosion score, (**B**) total backfill score, (**C**) total ankylosis score and (**D**) fat metaplasia

There were no significant between-group differences for total ankylosis (*P* = 0.46) or fat metaplasia (*P* = 0.17) (Fig. 1; Supplementary Table S3, available at *Rheumatology* online). These findings were supported by cumulative probability plots showing changes from baseline to week 12 in erosion, backfill, ankylosis and fat metaplasia (Fig. 3). There was little variation between the two independent MRI readers (Table 2). ICC values ranged from 0.69 (erosion) to 0.97 (ankylosis) at baseline and from 0.71 (erosion) to 0.95 (ankylosis) at week 12. For the change from baseline to week 12, ICC values were 0.76, 0.73 and 0.62 for erosion, backfill and fat metaplasia, respectively. The ICC value for the change from baseline in ankylosis was not applicable, as changes were not expected over a 12 week timeframe.

**Table 2:** Interreader ICCs for structural lesions

Category	Time point	ICC	95% CI
Erosion	Baseline	0.687	0.531, 0.790
	Week 12	0.710	0.569, 0.806
	Change from baseline to week 12	0.764	0.661, 0.838
Backfill	Baseline	0.703	0.593, 0.787
	Week 12	0.797	0.708, 0.861
	Change from baseline to week 12	0.733	0.620, 0.817
Fat metaplasia	Baseline	0.788	0.665, 0.863
	Week 12	0.799	0.694, 0.868
	Change from baseline to week 12	0.616	0.467, 0.731
Ankylosis	Baseline	0.972	0.959, 0.981
	Week 12	0.954	0.931, 0.969
	Change from baseline to week 12	0.002^a^	−0.201, 0.207

a Almost no change in ankylosis recorded by either reader. ICC here is not valid.

### Changes in structural lesions according to baseline SPARCC BMO score

Supplementary Table S4, available at *Rheumatology* online, shows the proportion of patients with structural changes that decreased, increased or did not change between baseline and week 12 for the filgotinib and placebo groups in the subgroup of patients with a SPARCC BMO score ≥2 at baseline. In this subgroup there was a significant treatment difference among the change categories for backfill. In the filgotinib group, backfill increased in 8/22 patients (36.4%) and decreased in 1/22 patients (4.5%), whereas in the placebo group, backfill did not increase in any patients but decreased in 3/22 patients (13.6%; *P* = 0.014). Treatment differences for erosion, ankylosis and fat metaplasia were not significant. There were no significant differences for any of the structural lesions in the subgroup of patients with a SPARCC BMO score <2 at baseline (Supplementary Table S5, available at *Rheumatology* online).

### Correlation between structural domain changes, inflammation and clinical parameters

Changes in erosion score at week 12 correlated positively with changes in the SPARCC MRI SI joint inflammation score in both the filgotinib group (*r* = 0.359, *P* = 0.013) and placebo group (*r* = 0.560, *P* < 0.001). A negative correlation was observed for backfill in both the filgotinib (*r* = −0.415, *P* = 0.004) and placebo (*r* = −0.375, *P* = 0.019) groups. No significant correlations were observed between erosion or backfill scores and other clinical endpoints. No significant correlations were observed between ankylosis or fat lesion scores with any of the clinical endpoints assessed.

### Proportion of resolved erosion associated with the development of backfill

According to individual reader data, erosion resolved in 8 patients (reader 1) and 11 patients (reader 2) in the filgotinib group and in no patients (reader 1) and 5 patients (reader 2) in the placebo group (resolution was defined as being present at baseline but not at week 12/early discontinuation at a particular location within the joint). Backfill developed in 54.2% (reader 1) and 57.3% (reader 2) of locations (SI joint quadrants) with resolved erosion in the filgotinib group and in 20.0% of the locations with resolved erosion reported by reader 2 in the placebo group (there were no cases of resolved erosion reported by reader 1) (Supplementary Table S6, available at *Rheumatology* online).

## Discussion

In this post hoc analysis of the 12 week, phase 2 TORTUGA trial, filgotinib, which preferentially inhibits JAK1, was associated with structure-modifying effects on MRI structural lesions in the SI joint of patients with AS. Compared with placebo, patients treated with filgotinib 200 mg had a significant decrease in SPARCC SSS erosion scores and a significant increase in SPARCC SSS backfill scores. No statistically significant between-group differences were observed between the mean change from baseline to week 12 in fat metaplasia or ankylosis score. Changes in erosion and backfill scores correlated significantly with changes in inflammation in the SI joint, as measured by the SPARCC MRI SI joint inflammation score, although the small patient numbers limit the interpretation of these findings. Only half of the resolved erosions were associated with backfill repair. Consistency was seen between readers, with interreader reliability comparable to that reported for a previous trial [18].

TORTUGA was the first clinical study to investigate a selective JAK1 inhibitor for the treatment of adult patients with active AS. As previously reported, the initial study met its primary endpoint, with filgotinib-treated patients showing decreased disease activity, including MRI-documented inflammation. SPARCC SI joint and spine inflammation scores decreased significantly (*P* = 0.0150 and *P* = 0.0066, respectively) *vs* placebo over 12 weeks [23]. The reduction in erosion and increased backfill observed in this post hoc analysis also occurred within the first 12 weeks of treatment and thereby seem to be directly associated with the resolution of inflammation. Our findings are suggestive of an early reparative response with backfill (i.e. the in-filling of an eroded cavity).

Similar effects have been observed with anti-TNF therapy [17, 18]. A post hoc analysis of the EMBARK trial, a randomized, phase 3b, placebo-controlled trial of etanercept in 185 patients with non-radiographic axSpA, showed a reduction in SPARCC SSS erosion (−0.49; *P* = 0.017) and an increase in SPARCC SSS backfill (0.30; *P* = 0.022) with etanercept after 12 weeks *vs* placebo [18]. The effect was particularly pronounced for patients with baseline SI joint inflammation on MRI. The decrease in erosion and increase in backfill correlated with an improvement in several clinical outcomes in patients receiving etanercept. Similarly, in a smaller placebo-controlled study involving 52 patients with r-axSpA and non-radiographic axSpA, SPARCC erosion scores decreased significantly whereas backfill scores increased numerically after 12 weeks of adalimumab treatment [17]. Development of backfill and fat lesions are correlated [13], so it was surprising to observe no impact of filgotinib on the development of new fat lesions. This could reflect the relatively small sample size, a 12 week primary endpoint that may be too short for fat lesions to be observed or even an impact of treatment. Further longitudinal data in patients on treatment are necessary.

The mechanisms by which JAK inhibitors might ameliorate inflammation and induce erosion repair (and how this influences new bone formation) remain to be elucidated. Many cytokines implicated in innate and adaptive immune responses underlie the pathogenesis of AS signal through JAK pathways [20]. Interferon-α, IL-6, IL-10, IL-15 and IL-22 signalling pathways are blocked directly by JAK1 inhibition, with the potential for indirect inhibition of TNF, IL-1 and IL-17 pathways [20]. Activation of these pathways in AS results in the proliferation of peri- and extra-articular inflammatory cells and cell types associated with bone loss and joint destruction.

Our study had some limitations. It was a post hoc analysis with small patient numbers. Half the patients demonstrated substantial ankylosis in the SI joint, limiting the capacity to demonstrate change in other structural features. No changes in total ankylosis score were expected during the relatively short timeframe (12 weeks) and given that ankylosis was generally at an advanced state at baseline. The impact of filgotinib, and JAK inhibitors in general, on structural lesions should be explored in longer-term studies.

Although MRI scans were unavailable for 29 patients, the population in the MRI substudy was generally similar to the overall population. In addition, missing MRIs were mainly due to patient-related factors (e.g. claustrophobia or deformation of the spine preventing the MRI from being performed) or logistical issues leading to MRI scans being obtained outside the protocol-defined time window, all of which occurred prior to unblinding of the study. In addition, an unpublished sensitivity analysis in the main TORTUGA trial, which included a broader set of out-of-window MRIs, revealed a similar outcome to the main analysis for the SPARCC SI joint endpoint. We therefore believe that, other than reducing the number of evaluable patients, the missing MRIs did not have a relevant impact on the interpretation of the data. Patients with MRI structural lesions, especially fat lesions and backfill, represent a subset with higher prognostic risk of progression—not just in the SI joint, but also the spine [30]. They are more likely to be male and HLA-B27 positive [7, 30]. It is of interest that only half of patients develop backfill when erosion decreases after resolution of inflammation. The prognostic trajectory of patients who develop backfill may be worse [29]. Consequently, highly effective treatment is needed for these patients to prevent the development of new inflammatory lesions and additional structural lesions. Further longitudinal analysis in larger datasets will be necessary.

In conclusion, in addition to previously reported decreases in SPARCC inflammation observed with filgotinib in AS during the TORTUGA trial, filgotinib was associated with a significant reduction in SPARCC SSS erosion and an increase in backfill as early as 12 weeks. Research is needed to confirm these findings in larger clinical trials, and the prognostic implications of the structural changes observed in this analysis for disease/ankylosis progression warrant further research. In particular, investigation is needed into how the early effects of treatment on structural lesions might influence future development of ankylosis of the SI joint, new bone formation in the spine and the impact on clinical and mobility outcomes and disability.

## Supplementary Material

keab543_Supplementary_Data
