# Supplementary material for: Impact of filgotinib on sacroiliac joint magnetic resonance imaging structural lesions at 12 weeks in patients with active ankylosing spondylitis (TORTUGA trial)
Source: Rheumatology (Oxford). 2021 Aug 5;61(5):2063–71. doi: 10.1093/rheumatology/keab543 (PMC9071516; doi:10.1093/rheumatology/keab543)
Supplement: keab543_Supplementary_Data [file keab543_supplementary_data.docx]

**Supplementary material for:**

Impact of filgotinib on sacroiliac joint MRI structural lesions at 12 weeks in patients with active ankylosing spondylitis (TORTUGA trial)

# Walter P. Maksymowych, Mikkel Østergaard, Robert Landewé, William Barchuk, Ke Liu, Chantal Tasset, Leen Gilles, Thijs Hendrikx, Robin Besuyen, Xenofon Baraliakos

**Corresponding author:**

Walter P. Maksymowych

Department of Medicine, University of Alberta 568 Heritage Medical Research Building Edmonton, Alberta T6R 2G8, Canada

Email: [walter.maksymowych@ualberta.ca](mailto:walter.maksymowych@ualberta.ca)

**SUPPLEMENTARY TABLE S1** Ethics committees

| **Site** | **Ethics committees** |
| --- | --- |
| CHU Sart Tilman, University of Liège, Liège, Belgium | Comité d’Ethique Hospitalo-Facultaire Universitaire de LiègeCommissie voor Medische Ethiek |
| Erasme Hospital, Brussels, Belgium | Comité d'Ethique CUB Hôpital Erasme Commissie voor Medische Ethiek |
| VIB Center for Inflammation Research, Ghent, Belgium | Commissie voor Medische Ethiek |
| CHU Ambroise Paré, Mons, Belgium | Comité d'Ethique du CHU Ambroise Paré Commissie voor Medische Ethiek |
| UMHAT "Kaspela", EOOD, Plovdiv, Bulgaria | Ethics Committee for Multicenter Trials |
| UMHAT St. Ivan Rilski, Sofia, Bulgaria | Ethics Committee for Multicenter Trials |
| MHAT - Ruse, Ruse, Bulgaria | Ethics Committee for Multicenter Trials |
| UMHAT "Kaspela", EOOD, Plovdiv, Bulgaria | Ethics Committee for Multicenter Trials |
| Second Internal Clinic UMHAT Stara Zagora, Stara Zagora, Bulgaria. | Ethics Committee for Multicenter Trials |
| CCR Czech a.s., Pardubice, Czech Republic | Eticka komise Fakultni nemocnice Kralovske Vinohrady |
| Revmatologicka ambulance, Nusle, Czech Republic | Eticka komise Fakultni nemocnice Kralovske Vinohrady |
| University of Veterinary and Pharmaceutical Sciences Brno, Brno, Jihomoravský, Czech Republic | Eticka komise Fakultni nemocnice Kralovske Vinohrady |
| North Estonian Medical Centre, Tallinn, Estonia | Tallinn Medical Research Ethics Committee |
| East Tallinn Central Hospital, Tallinn, Estonia | Tallinn Medical Research Ethics Committee |
| Klinische Forschung Berlin, Berlin, Germany | Ethik-Kommission der medizinischen Fakultät der Albert-Ludwigs-Universität Freiburg i.Br. |
| University of Freiburg, Freiburg, Germany | Ethik-Kommission der medizinischen Fakultät der Albert-Ludwigs-Universität Freiburg i.Br. |
| Krankenhaus St. Josef, Wuppertal, Germany | Ethik-Kommission der medizinischen Fakultät der Albert-Ludwigs-Universität Freiburg i.Br. |
| University Hospital Mainz, Mainz, Germany | Ethik-Kommission der medizinischen Fakultät der Albert-Ludwigs-Universität Freiburg i.Br. |
| Charité-Universitätsmedizin Berlin, Berlin, Germany | Ethik-Kommission der medizinischen Fakultät der Albert-Ludwigs-Universität Freiburg i.Br. |
| Centrum Medyczne AMED, Warsaw, Poland | Komisja Bioetyczna przy WIL |
| Twoja Przychodnia Centrum Medyczne Nowa Sol, Nowa Sol, Poland | Komisja Bioetyczna przy WIL |
| ClinicMed Daniluk, Białystok, Poland | Komisja Bioetyczna przy WIL |
| Jan Biziel University Hospital No. 2, Bydgoszcz, Poland | Komisja Bioetyczna przy WIL |
| Szpital Specjalistyczny, Bytom, Poland | Komisja Bioetyczna przy WIL |
| Wroclaw Medical University, Wrocław, Poland | Komisja Bioetyczna przy WIL |
| National Institute of Geriatrics, Rheumatology and Rehabilitation, Warsaw, Poland | Komisja Bioetyczna przy WIL |
| Ai Centrum Medyczne sp. z o.o. sp.k, Poznan, Poland | Komisja Bioetyczna przy WIL |
| Universidad de Málaga, Málaga, Spain | CEIC Hospital Universitario Madrid Monteprincipe |
| Hospital Universitari Parc Taulí Sabadell, Medicine Department UAB, Barcelona, Spain | CEIC Hospital Universitario Madrid Monteprincipe |
| Hospital Clínico Universitario de Santiago, Instituto de Investigación Sanitaria de Santiago (IDIS), Santiago de Compostela, Spain | CEIC Hospital Universitario Madrid Monteprincipe |
| Hospital Fuenlabrada, Fuenlabrada, Spain | CEIC Hospital Universitario Madrid Monteprincipe |
| Hospital Universitario Virgen Macarena, Sevilla, Spain | CEIC Hospital Universitario Madrid Monteprincipe |
| Communal Establishment of Health Protection "Kharkiv City Clinical Hospital #8", Kharkiv, Ukraine | LEC Communal Institution of Healthcare Kharkiv City Clinical Hospital #8 |
| M.V.Sklifosovskyi Poltava Regional Clinical Hospital, Poltava, Ukraine | LEC Poltava Reg.Clin.Hosp.n.a.M.V.Sklifosovskogo |
| National Pirogov Memorial Medical University, Vinnytsia, Ukraine | LEC Vinnytsia M.I.Pyrogov Regional Clinical Hospital  LEC Scient.&Research Instit.of Invalid Rehabiltation of Vinnytsia M.I.Pyrogov Nat.Med.Univer. |
| Medical Clinical Investigational Center of Medical Center Health Clinic LLC, Vinnytsia, Ukraine | LEC Med.Cl.Invest.Center Health Clinic |
| SI NSC M.D. Strazhesko Institute of Cardiology, NAMS of Ukraine, Kiev, Ukraine | LEC SI National Scientific Center Acad. M.D.Strazhesko |
| Danylo Halytsky Lviv National Medical University, Lviv, Ukraine | LEC CH of State Border Service of Ukraine (Military Base 2522) |
| Railway Clinical Hospital of Uzhorod Station of Lviv Railroad Administration, Uzhorod, Ukraine | LEC Zakarpatian Regional Clin.Hosp. n.a.A.Novak |
| Consult-Diagnostic Center of Pecherskyi District of Kyiv, Kyiv, Ukraine | LEC CNCE Consult-Diagnostic Center of Pecherskyi District of Kyiv |
| I. Horbachevsky Ternopil National Medical University, Ternopil, Ukraine | LEC Ternopil Regional Council Ternopil University Hospital |

**SUPPLEMENTARY TABLE S2** Disease activity in patients with and without MRI scans

| **Characteristic** | **Evaluable patients with MRI scans** | | **Excluded patients without MRI scans^a^** | | **Total population** | |
| --- | --- | --- | --- | --- | --- | --- |
|  | ***n*** | **Mean (S.D.)** | ***n*** | **Mean (S.D.)** | ***N*** | **Mean (S.D.)** |
| ASDAS | 87 | 4.2 (0.64) | 29 | 4.2 (0.89) | 116 | 4.2 (0.71) |
| MASES | 69 | 4.7 (2.82) | 26 | 4.0 (2.87) | 95 | 4.5 (2.83) |
| Swollen joint count 44 | 29 | 1.9 (1.60) | 6 | 2.5 (1.97) | 35 | 2.0 (1.65) |
| Tender joint count 44 | 70 | 4.5 (4.07) | 18 | 4.9 (2.84) | 88 | 4.6 (3.84) |
| BASDAI | 87 | 7.0 (1.23) | 29 | 6.9 (1.29) | 116 | 7.0 (1.24) |
| BASMI | 85 | 5.1 (1.65) | 29 | 5.5 (1.38) | 114 | 5.2 (1.60) |

^a^Excluded patients were those without an evaluable MRI scan at both baseline and week 12 or early discontinuation visit. ASDAS: Ankylosing Spondylitis Disease Activity Score; MASES: Maastricht Ankylosing Spondylitis Enthesitis Score.

# **SUPPLEMENTARY TABLE S3** Summary of Spondyloarthritis Research Consortium of Canada Sacroiliac Joint Structural Scores

| **Score** | **Mean (S.D.) baseline score** | **LSM change from baseline**  **(95% CI) at**  **week 12** | **LSM group difference at week 12 (95% CI)**  **[*P-*value]** |
| --- | --- | --- | --- |
| Erosion |  |  |  |
| Filgotinib 200 mg | 3.38 (5.34) | −0.46 | −1.01 |
|  |  | (−1.31, 0.40) | (−1.87, −0.16) |
| Placebo | 2.62 (3.76) | 0.56 | [*P* = 0.02] |
|  |  | (−0.31, 1.42) |  |
| Backfill |  |  |  |
| Filgotinib 200 mg | 1.02 (1.99) | 0.76 | 1.02 |
|  |  | (0.07, 1.45) | (0.32, 1.72) |
| Placebo | 1.35 (2.59) | −0.26 | [*P* = 0.005] |
|  |  | (−0.97, 0.45) |  |
| Fat metaplasia |  |  |  |
| Filgotinib 200 mg | 4.19 (6.06) | 0.37 | 0.43 |
|  |  | (−0.23, 0.97) | (−0.18, 1.03) |
| Placebo | 4.35 (5.44) | −0.06 | [*P* = 0.17] |
|  |  | (−0.67, 0.56) |  |
| Ankylosis |  |  |  |
| Filgotinib 200 mg | 9.58 (8.15) | 0.14 | 0.06 |
|  |  | (−0.02, 0.30) | (−0.10, 0.22) |
| Placebo | 9.83 (8.45) | 0.08 | [*P* = 0.46] |
|  |  | (−0.08, 0.25) |  |

LSM: least-squares mean.

# **SUPPLEMENTARY TABLE S4** Proportion of patients with a decrease, increase or no change^a^ in structural lesion score at week 12, in the subgroup with baseline SPARCC BME score ≥2

| **Change between baseline and week 12, *n* (%)** | | | | | |
| --- | --- | --- | --- | --- | --- |
| **Lesion** | **Treatment** | **Decrease** | **No change** | **Increase** | ***P-*value**^b^ |
| Erosion | Filgotinib (*n* = 22) | 8 (36.4) | 12 (54.5) | 2 (9.1) | 0.066 |
|  | Placebo (*n* = 22) | 2 (9.1) | 17 (77.3) | 3 (13.6) |  |
| Backfill | Filgotinib (*n* = 22) | 1 (4.5) | 13 (59.1) | 8 (36.4) | 0.014 |
|  | Placebo (*n* = 22) | 3 (13.6) | 19 (86.4) | 0 |  |
| Ankylosis | Filgotinib (*n* = 22) | 0 | 22 (100) | 0 | – |
|  | Placebo (*n* = 22) | 0 | 22 (100) | 0 |  |
| Fat metaplasia | Filgotinib (*n* = 22) | 1 (4.5) | 16 (72.7) | 5 (22.7) | 0.119 |
|  | Placebo (*n* = 22) | 0 | 22 (100) | 0 |  |

^a^A lesion was considered to have decreased or increased when both readers categorised the change in the same direction (i.e. both reported either a decrease or increase). If one reader categorised the change as 0 or if readers categorised a change in opposite directions (i.e. one reported a decrease and one reported an increase), then no change was chosen. ^b^*P*-values are based on an ordered logistic regression model. BME: bone marrow oedema; SPARCC: Spondyloarthritis Research Consortium of Canada.

# **SUPPLEMENTARY TABLE S5** Proportion of patients with a decrease, increase or no change^a^ in structural lesion score at week 12, in the subgroup with baseline SPARCC BME score <2

| **Change between baseline and week 12, *n* (%)** | | | | | |
| --- | --- | --- | --- | --- | --- |
| **Lesion** | **Treatment** | **Decrease** | **No change** | **Increase** | ***P-*value**^b^ |
| Erosion | Filgotinib (*n* = 26) | 1 (3.8) | 25 (96.2) | 0 | 0.946 |
|  | Placebo (*n* = 17) | 0 | 17 (100) | 0 |  |
| Backfill | Filgotinib (*n* = 26) | 0 | 26 (100) | 0 | – |
|  | Placebo (*n* = 17) | 0 | 17 (100) | 0 |  |
| Ankylosis | Filgotinib (*n* = 26) | 0 | 26 (100) | 0 | – |
|  | Placebo (*n* = 17) | 0 | 17 (100) | 0 |  |
| Fat metaplasia | Filgotinib (*n* = 26) | 1 (3.8) | 22 (84.6) | 3 (11.5) | 0.567 |
|  | Placebo (*n* = 17) | 2 (11.8) | 13 (76.5) | 2 (11.8) |  |

^a^A lesion was considered to have decreased or increased when both readers categorised the change in the same direction (i.e. both reported either a decrease or increase). If one reader categorised the change as 0 or if readers categorised a change in opposite directions (i.e. one reported a decrease and one reported an increase), then no change was chosen. ^b^*P*-values are based on an ordered logistic regression model. BME: bone marrow oedema; SPARCC: Spondyloarthritis Research Consortium of Canada.

# **SUPPLEMENTARY TABLE S6** Mean percentages of locations (SI joint quadrants) with resolved erosion, with or without backfill, in patients with resolved erosion

| **Reader 1** | | | | **Reader 2** | |
| --- | --- | --- | --- | --- | --- |
|  |  | **Number of patients with resolved erosion** | **Mean percentage^a^ (S.D.) of**  **locations with**  **resolved erosion** | **Number of patients with resolved erosion** | **Mean percentage^a^ (S.D.) of**  **locations with**  **resolved erosion** |
| With developing backfill | Filgotinib 200 mg | 8 | 54.2 (42.49) | 11 | 57.3 (47.77) |
|  | Placebo | - | - | 5 | 20.0 (44.72) |
| Without backfill | Filgotinib 200 mg | 8 | 45.8 (42.49) | 11 | 42.7 (47.77) |
|  | Placebo | - | - | 5 | 80.0 (44.72) |

Developing backfill was defined as a location (SI joint quadrant) with no backfill at baseline and with backfill at week 12 or early discontinuation visit. Resolved erosion was defined as a location (SI joint quadrant) with erosion at baseline and no erosion at week 12 or early discontinuation visit. There were 13 and 22 SI joint quadrants with resolved erosion for reader 1 and reader 2, respectively. ^a^The percentages of locations (SI joint quadrants) with resolved erosion, which developed/resolved backfill was calculated for each patient with erosion resolution in at least one location. The mean of these percentages over the number of patients with resolved erosion was then presented as the mean percentage.
